# Measurement of the percentage of root filling in oval-shaped canals obturated with Thermafil Obturators and Beefill 2in1: In vitro study

**DOI:** 10.4317/jced.52177

**Published:** 2015-04-01

**Authors:** Vicente Faus-Llácer, Nicolás Collado-Castellanos, Teresa Alegre-Domingo, María Dolz-Solsona, Vicente Faus-Matoses

**Affiliations:** 1 DDS, Professor. Department of Dental Pathology, Conservative Dentistry and Endodontic, Department of Stomatology, Faculty of Medicine and Odontology, University of Valencia; 2 Postgraduate Degree. Department of Dental Pathology, Conservative Dentistry and Endodontic, Department of Stomatology, Faculty of Medicine and Odontology, University of Valencia; 3 Associate Professor. Department of Dental Pathology, Conservative Dentistry and Endodontic, Department of Stomatology, Valencia University Medical and Dental School

## Abstract

**Background:**

The aim of the study was to measure the percentage of root canal fillings in long oval canals obturated with thermoplasticized gutta-percha techniques, Beefill 2in1® and Thermafil Obturators®.

**Material and Methods:**

Fifty four mandibular incisors were selected after bucco-lingual and mesio-distal radiographs showed at 5 mm from apex an internal long:short diameter ≥2. Teeth were instrumented with Protaper Universal and divided in two groups of 27. Group 1 was obturated with Thermafil Obturators® and group 2 with Beefill 2in1®. Two horizontal sections were cut at 5 and 7 mm from the apex and photographed in a stereo-microscope. The total area of the canal and filled canal in cross-sections were measured with AutoCad and the percentages of gutta-percha-sealer and voids in the canal were obtained.

**Results:**

Both systems achieved high percentage of filled canal, Thermafil 96.8% and Beefill 2in1 98.9%. The percentages of voids in both groups were very low. No significant differences were found between the two groups . The percentage obtained at 5 and 7 mm from the apex in both groups showed no significant difference.

**Conclusions:**

The percentages of filled canal (gutta-percha-sealer) were high and these two thermoplasticized techniques are suitable for long oval canals obturation.

** Key words:**Long oval canal, oval canal, thermoplasticized obturation.

## Introduction

Radicular canals have different shapes in cross-sections. Oval-shaped canals were observed in 25% of teeth at 5 mm from apex (1). An oval canal was identified when the large diameter was higher than the small diameter (2).

Instrumentation and three-dimensional obturation of these canals could be a challenge for dentists. Many investigations had found this canal shape may difficult chemical-mechanical preparation of radicular canal; both manual instrumentation techniques and rotatory techniques left uninstrumented buccal and lingual extensions (3-5). The middle and coronal thirds of the preparation were more poorly instrumented due to the fact that the files did not make contact with all canal walls, according to the width of the canal (6,7). The quality of root canal fillings may be negatively influenced by the irregular canal shape and inadequate debridement (8).

To assess the quality obturation of oval canals, different methods had been described: radiographic assessment (9), fluid transport and polymicrobial leakage (8-12), and cross-sections of filled canal roots. Using cross-sections, the filled area was measured regarding total canal area and it allowed to calculate the percentage of gutta-percha (PGP) (8,9,13) or the percentage of guttapercha and sealer (3,12,14). These investigations have related the quality of root fillings with the gutta-percha-filled area or gutta-percha-sealer-filled area.

PGP obtained with lateral condensation (LC) in oval canals were widely studied in the literature, the values were ranged from 55% to 98.8% (3,8,9). However, Thermafil® obturation system (Dentsply Maillefer, Ballaigues, Switzerland) reported higher PGP and more predictable results than LC, because thermoplasticized gutta-percha had better adjustment qualities to the radicular canals (12-14).

More recently, micro-computed tomography (µCT) was used to quantify the percentage of filling materials and voids presented in oval shaped canals with cold and warm gutta-percha obturation techniques, reporting a low percentage of voids (15).

The sealing capability of Beefill 2in1® was measured by micro-leakage studies in round shape canals (16,17), but not in oval shaped canals. The literature did not report PGP using Beefill 2in1®; for this reason, the aim of this study was to compare the percentage of filled root canal (gutta-percha and sealer) in long oval canals, using Beefill 2in1® and Thermafil Obturators®.

## Material and Methods

-Sample selection

106 single-rooted mandibular incisors were used. Single oval-shaped canal morphology was confirmed by two radiographs made in a bucco-lingual and mesio-distal direction. Teeth with single canal and ratio of long: short diameter ≥2 at 5 mm from the apex were selected. Teeth with accessory or two canals were excluded. A total sample of 54 teeth were stored in distilled water.

-Instrumentation

After access opening to the root canal system, size 10 K-file was introduced to length in the canal space and the working length was established at 1 mm from the apical foramen by visual observation. The patency of each canal was confirmed by inserting a size 15 file through apical foramen. All canals were prepared with Protaper Universal (Dentsply Maillefer, Ballaigues, Switzerland) according to manufacturer instructions. Shaping files S1 and S2 were used with a brushing and circumferential motion to the working length, then Finishing files F1, F2 and/or F3 were used with pecking motion 250 rpm and 3 Ncm using X-Smart engine (Dentsply Maillefer, Ballaigues, Switzerland). After using of each file the canal was irrigated with 2% NaOCl. When the canals were prepared, in order to eliminate the smear layer, a final flush of 1 mL of 18% EDTA (Ultradent, Koln, Germany) for 1 min was performed and was washed with 2mL of 2% NaOCL. The canals were dried with F2 or F3 paper points (Dentsply Maillefer, Ballaigues, Switzerland).

-Obturation

The teeth were randomly divided into two groups. TopSeal (Dentsply Maillefer, Ballaigues, Switzerland) sealer was introduced into canals to working length with a F2 or F3 gutta-percha point (Dentsply Maillefer, Ballaigues, Switzerland) according to apical diameter.

Group 1: 27 teeth were obturated with Thermafil obturators F2 or F3 (Dentsply Maillefer, Ballaigues, Switzerland) heated in Thermaprep Plus oven (Dentsply Maillefer, Ballaigues, Switzerland) in accordance with manufacturer’s recommendations. Thermafil obturators were inserted into the canals to working length. A rounded bur was used to cut the carriers when the gutta-percha was cold.

Group 2: 27 teeth were obturated with Protaper F2 or F3 gutta-percha point using Beefill 2in1® device (VDW, München, Germany) with continuous wave technique (WCV). Gutta-percha point was fitted 0.5 mm short of the working length with tug-back. The Beefill Downpack device was used to obturate the apical third of root canal and it was pre-set at 180ºC, then a hot plugger was introduced 5 mm short from the working length. After that a plugger 1-2 (Maillefer, Ballaigues, Switzerland) was used to condensate the apical master cone gutta-percha. Middle and coronal thirds of the root canal were filled using a Beefill Backfill device set at 180ºC and downpacking with the plugger.

After all teeth were obturated, they were stored for 7 days at 100% humidity before preparing the samples.

-Sample preparation and analysis

The teeth were cut horizontally 5 and 7 mm from the apical foramen using a low hand-piece with a 0.3 mm thick diamond disc (Komet, Lemgo, Germany) with constant water irrigation.

Cross-sections were embedded in putty silicone in order to improve their manipulation and observation. Surfaces were dried and photographed under standardized conditions at 4.5x magnification using a digital camera attached to a stereomicroscope (Novex, BD Arnhem, Holland).

Image analysis was carried out by using AutoCad 2006 for Windows XP (Autodesk Inc, San Rafael, California). The cross-section area of the canal and the filled canal area (gutta-percha + sealer) were measured and the percentage of filled canal and voids were calculated.

The data for the percentages were analyzed descriptively and analyzed statistically using the Mann-Whitney U-test. The level of significance was set at *P* < 0.05.

## Results

53 cross-sections for group 1 and 54 for group 2 were obtained. One transversal section of Thermafil group was rejected because the image was not able to analyze.

The mean percentage of filled canal for group 1 was 96.8% and for Beefill 2in1 group was 98.9%, there were no significant differences between the two groups (*P* > 0.05). 50.9% of Thermafil sample obtained a total canal obturation against 61.1% of Beefill 2in1. We observed a greater dispersion of the results of group 1 compared to group 2, in group 1 the percentages ranged from 67% to 100%, in contrast in group 2 ranged from 89.5% to 100%.

The percentages of canal filled and voids Table 1. Both for Thermafil group and Beefill 2in1 group no statistically significant differences were observed (*P* > 0.05) depending on distance from the apex.

**Table 1:**
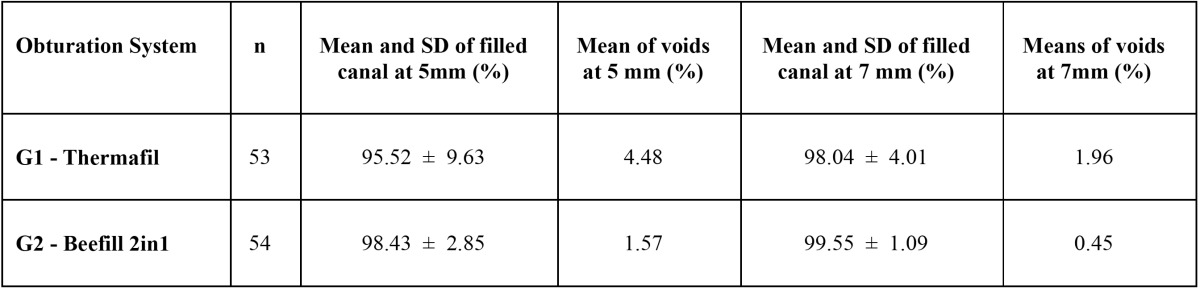
Percentages of samples according to sealing system and distance from the apex.

## Discussion

Long oval canals have a large diameter at least two times greater than the short diameter, being the bucco-lingual diameter longer than the mesio-distal (1). In this study long oval canals were filled with two thermoplasticized canal obturation techniques. Ther-mafil Obturators and Beefill 2in1 showed high percentages of filled canal and there were not significant differences between them. By means of analysing cross-sectioned roots is possible to calculate the percentage of filled canal and voids in order to provide the quality of canal filling (18). Following the approach described by Gençoglu *et al.* (19), the samples were photographed under magnification of a stereomicroscope and were analyzed with AutoCad (Autodesk Inc, San Rafael, California), to calculate the area occupied by gutta-percha-sealer and the area of voids.

**Figure 1:**
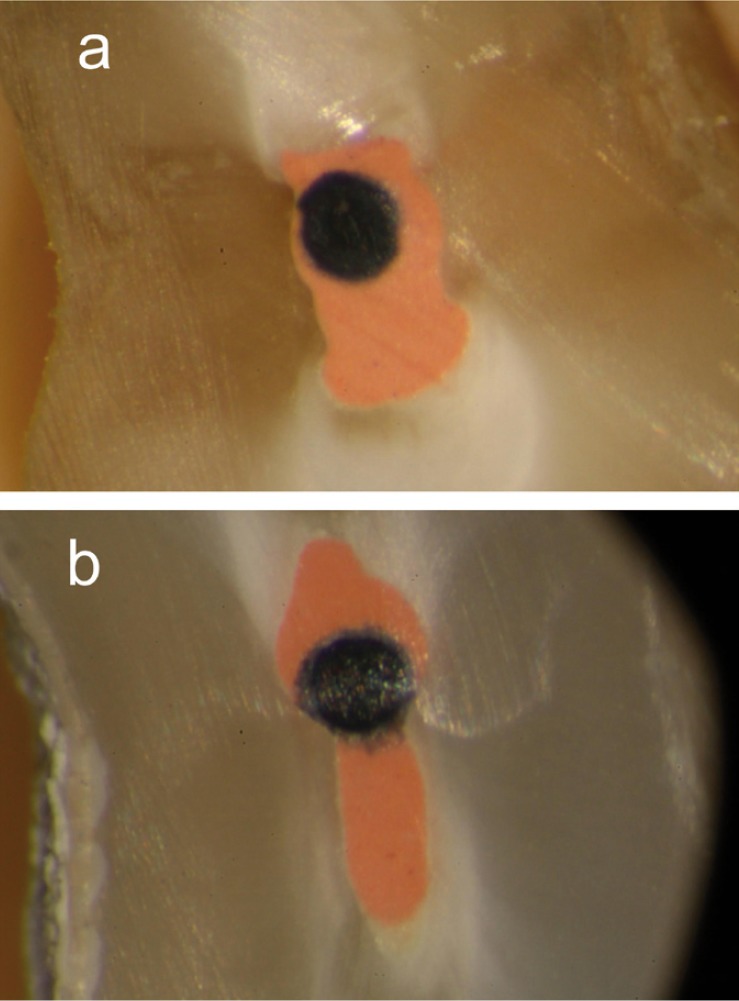
Cross-sections of group 1. a) Cross-section at 5 mm from the apex. All canals were completely prepared and obturated. b) Cross-section at 7 mm from apex, the recess was obturated with gutta-percha and amount of sealer.

There is not a defined protocol in the literature to evaluate oval canals filling, with regard to the level of cross-section, the use of sealer or not and the removal of smear layer. In the present study, group 1 obtained a mean PGP value higher than 95% (Fig. 1), which is in agreement with Ozawa et al. (14). In contrast, the present results are not in agreement with some investigations; mean values were reported lower than 90% at 5mm from the apex (12,13). These differences can be explained by some methodologic variations. In one study the root canals were instrumented using the balanced force technique (12); and De-Deus *et al.* (13) did not use any irrigant solution to eliminate the smear layer and neither any root canal sealer was used. Manual preparation has been reported to leave more uninstrumented recesses in buccal and lingual aspects compared to rotatory techniques (20). In the present study, oval-shaped canals were prepared with Protaper Universal (Denstply, Maillefer, Ballaigues, Switzerland). ElAyouti *et al.* (5) found a better ratio of prepared oval root canal walls with Protaper files than with NiTi- hand files and Hedström and also compared with Hero instruments. Moreover, in one study, distal oval canals of mandibular molars were instrumented using Protaper Universal (Denstply, Maillefer, Ballaigues, Switzerland) considering the oval canal as a unique canal or two canals; they concluded that preparation oval canals as two separated entities may be beneficial to get a lower unprepared canal surface, regardless of instrumentation technique used (21).

**Figure 2:**
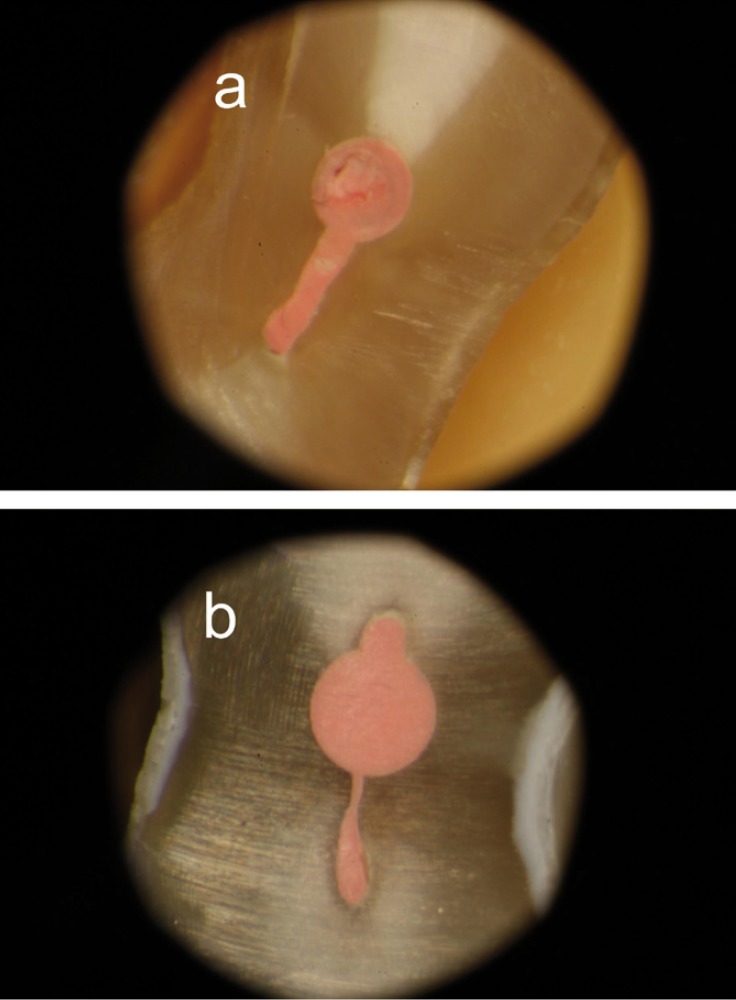
Representative specimen of group 2. a) Cross-section at 5 mm from the apex. b) Cross-section at 7 mm from the apex. In both images the recess was completely filled.

In group 2 (Fig. 2), the mean percentage of canal filled at 5mm from the apex was 98.4%, in concordance with Wu *et al.* (8); they carried out a warm vertical condensation through the Touch n’ Heat and injection of warm gutta-percha with the Hygenic Ultrafil system. Similar results (91.8%) were obtained in round shape canals at 2 and 4 mm from the apex (22). These studies had some differences in the methodology to obtain the cross-sections compared with the present study; thus the sections were got at 5 mm from the apex as the major percentage of long oval canals reported in mandibular incisors were found at 5 mm from the apex, where the long:short diameter ratio was ≥ 2x (1).

In a recent study, better results were observed using Beefill 2in1® than “single-cone technique” in terms of less voids per tooth and microleakage. In addition, in the Beefill 2in1 group the master cone was better fitted to the radicular canal even when an oval canal was presented compared with the single-cone technique (23). In accordance with this study it was observed that radicular canals filled by a central mass of gutta-percha, and a very low percentage of voids due to the warm vertical condensation of the gutta-percha cone. Nowadays, with the advantages of the µ-CT, filled root canals could be evaluated in three dimensions and the samples could be preserved along the study without irreversible structural damage. Mean percentage of gutta-percha and sealer was 99.43% using the warm vertical compaction technique, in agreement with this study (15).

**Figure 3:**
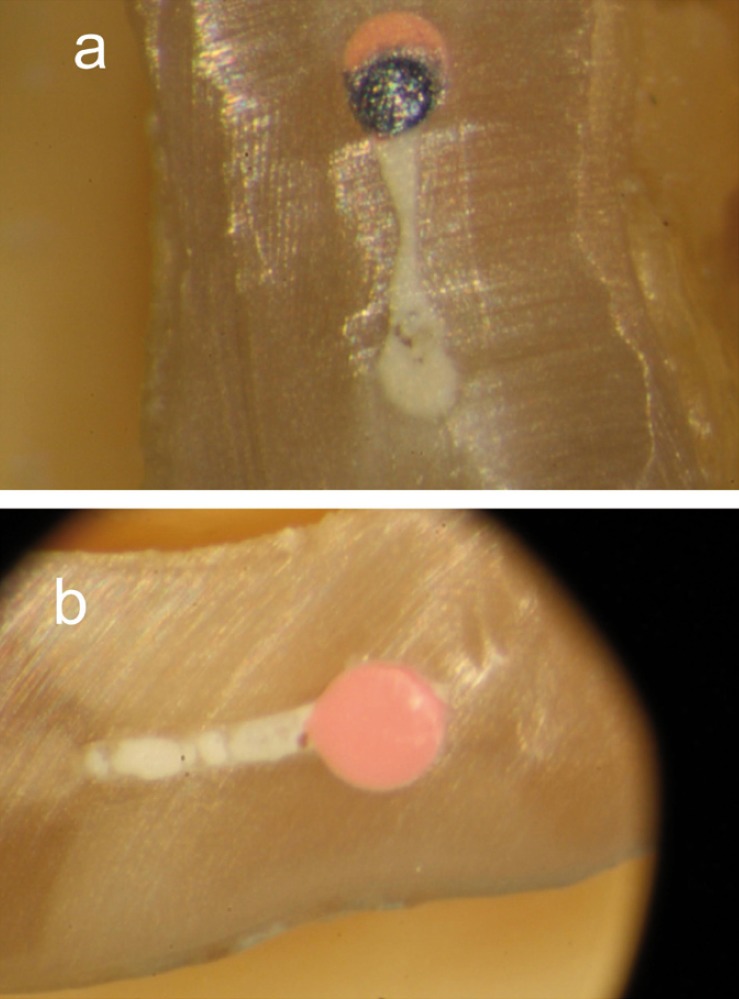
Cross-sections of both groups at 5 mm from the apex. a) Thermafil section showed the recess filled with voids and a big amount of sealer. b) Beefill 2in1 section showed the recess filled with some voids and sealer.

In this study, the percentages of filled canal at 7 mm from the apex were higher than at 5 mm in both groups. It may depend on width, length and cleanliness of buccal-lingual recesses (3) and the fact that in narrow canals the accessibility of the rotatory instruments to the whole outline is limited (5). In most of the cross-sections it was observed that canals were incompletely prepared, the buccal and lingual extensions remained uninstrumented and round shape was found in the center of the root (Figs. 1,2), in accordance with the results of Rödig et al. (6). 22 samples (20.56%) showed buccal and/or lingual extensions filled with a large amount of sealer and no gutta-percha was found in them (Fig. 3). This finding was in contrast with Wu et al. (24), the sealer should be allocated in a thin layer between the gutta-percha and the walls of the canal. This fact avoids the leakage due to shrinkage and dissolution of cement.

To conclude, the obturation of long oval canals with thermoplasticized techniques, Thermafil Obturators® and Beefill 2in1®, achieved high percentages of filled canal. Although, bucal/lingual extensions of oval canals remained unprepared, the two systems obtained satisfactory results with low percentage of voids.
